# Reply to letter: The Effect of Biofeedback Therapy on ICIQ-SF Scores and Urodynamic Parameters

**DOI:** 10.5812/numonthly.4270

**Published:** 2012-06-20

**Authors:** Omer Bayrak, Ilker Seckiner

**Affiliations:** 1 University of Gaziantep, School of Medicine, Department of Urology, Gazıantep, Turkey

**Keywords:** Therapeutics, Pelvic Floor

Dear Editor,

We appeciate having the opportunity to reply to the letter regarding our article entitled, “The Effect of Biofeedback Therapy on ICIQ-SF Scores and Urodynamic Parameters in Patients with Stress Urinary Incontinence (SUI)”.

Biofeedback and pelvic floor muscle exercises are advised, especially for the treatment of stress incontinence and mixed incontinence, because they are low-cost and do not have any potential side-effects (1). However, biofeedback therapy alone, without pelvic base muscle exercises, is not enough for the treatment of SUI. We have determined that pelvic floor muscle exercises, repeated at home make a very positive and important contribution to treatment, especially when biofeedback therapy is applied in the hospital as well as. We emphasize that all patients should continue these exercises at home after their therapy at the hospital has ended, so that their current healing status can be maintained and possibly even increased.

Seckiner *et al.* found that higher maximal detrusor pressure levels were moderately correlated with higher ICIQ-SF scores. They concluded from their findings that the ICIQ-SF questionnaire may provide predictive information regarding urodynamic study results in an over-active bladder (2). In our study, patients were hesitant, because although urodynamic testing is not a difficult treatment, it is an invasive examination and has a risk of infection. Because of these reasons we think that a urodynamic test is not necessary for all patients who are planning to do biofeedback therapy. However, if surgical treatment is planned, urodynamic studies can be useful.
